# A DFT Study on the Molecular Mechanism of Additions of Electrophilic and Nucleophilic Carbenes to Non-Enolizable Cycloaliphatic Thioketones †

**DOI:** 10.3390/molecules26185562

**Published:** 2021-09-13

**Authors:** Grzegorz Mlostoń, Karolina Kula, Radomir Jasiński

**Affiliations:** 1 Department of Organic and Applied Chemistry, University of Łódź, Tamka 12, PL-91-403 Łódź, Poland; 2 Institute of Organic Chemistry & Technology, Cracow University of Technology, Warszawska 24, PL-31-155 Krakow, Poland; kkula@chemia.pk.edu.pl

**Keywords:** carbenes, thioketones, organic reactions mechanisms, DFT calculations, molecular electron density theory

## Abstract

The molecular mechanisms of addition of dihalocarbenes and dimethoxycarbene to thioketones derived from 2,2,4,4-tetrmethylcyclobutane-1,3-dione were examined on the basis of the DFT wb97xd/6-311g(d,p)(PCM) calculations. Obtained results demonstrated that the examined processes exhibit polar nature and in the case of electrophilic dichloro-, and dibromocarbenes are initiated by the attack of carbene species onto the sulfur atom of the C=S group. Remarkably, reactions involving more electrophilic carbenes (dichloro-, and dibromocarbene) proceeds via stepwise mechanism involving thiocarbonyl ylide as a transient intermediate. In contrast, analogous reactions with nucleophilic dimethoxycarbene occur via a single step reaction, which can be considered as the [2 + 1] cycloaddition reaction initiated by the attack onto the C=S bond. A computational study showed that difluorocarbene tends to react as a nucleophilic species and resembles rather dimethoxycarbene and not typical dihalocarbene species. Significantly higher reactivity of the thioketone unit in comparison to the ketone group, both present in 3-thioxo-2,2,4,4-tetramthylcyclobutanone molecule, was rationalized in the light of DFT computational study.

## 1. Introduction

Carbenes form a group of reactive intermediates in which the central the two-valent carbon atom plays the most important role, determining their structure and chemical behavior [1,2,3,4]. In the last 25 years, along with better known ‘classic’ electrophilic carbenes, the chemistry of nucleophilic analogues, and especially ‘nucleophilic heterocycloc carbenes’ (NHCs), has been under rapid development [5,6,7]. Great importance of both electrophilic and nucleophilic carbenes, in the current organic and organometallic chemistry, is demonstrated by a large number of original publications and reviews, which have appeared in the last five years [8,9,10,11,12]. Very recently, new reactions within situ generated carbenes, using non-conventional techniques, such as mechanochemical synthesis [13] or flow-technique [14], have also been reported.

**Figure 1:**
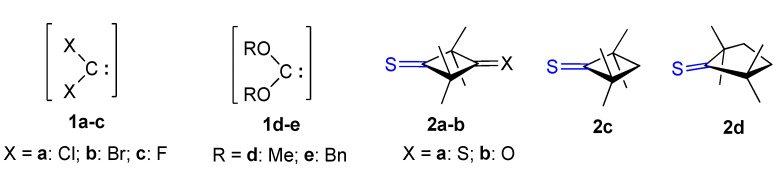
Dihalocarbenes, dialkoxycarbenes, and non-enolizable, cycloaliphatic thioketones **2a**–**d** involved in the study.

Dihalocarbenes **1a–b** [15] (Figure 1) occupy a prominent position in the group of electrophilic carbenes, and the Makosza reaction, based on the phase-transfer methodology, offers a straightforward access to synthetically relevant gem-dihalocyclopropanes via their [2 + 1] cycloaddition onto ethylenic >C=C< bonds [16,17,18,19]. In addition, difluorocarbene (**1c**) is considered an important building block for preparation of fluoroorganic compounds, which are of great importance for medicinal chemistry and related applications [20,21]. Among non-heterocyclic, nucleophilic carbenes, dialkoxycarbenes, such as dimethoxycarbene (**1d**) and dibenzyloxycarbene (**1e**), found numerous applications in organic synthesis and served as practically useful models for structural studies [22] (Figure 1).

**Scheme 1:**
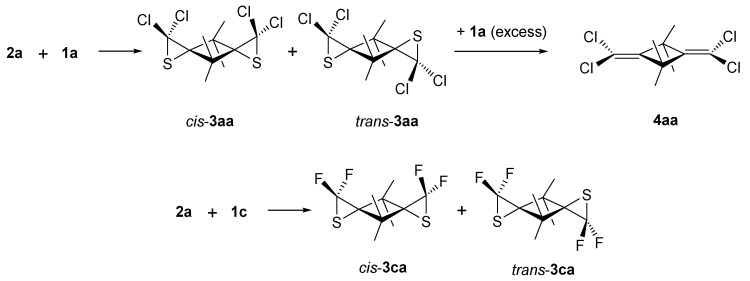
Products obtained in reactions of 2,2,4,4-tetramethylcyclobutane-1,3-dithione (**2a**) with dichlorocarbene (**1a**) and difluorocarbene (**1c**).

In our ongoing studies, attention has been focused on reactions of carbenes (and carbenoids) with lesser studied thiocarbonyl compounds and the non-enolizable thioketones being the favorite models. Depending on the type of carbenes used in the reaction with thioketones, formation of different products was observed. For example, in reactions of sterically crowded 2,2,4,4-tetramethylcyclobutan-1,3-dithione (**2a**) with difluorocarbene (**1c**) and dichlorocarbene (**1a**), generated either from Seyferth regaent (PhHgCX_3_) or under conditions of the two-phase Makosza rection (CHX_3_/NaOH/TEBA), formation of mixtures of isomeric spiro-gem-difluorothiirane *cis*-**3ca**, and *trans*-**3ca** or spiro-gem-dichlorothiirane *cis*-**3aa**, and *trans*-**3ba**, respectively, was observed (Scheme 1) [23,24]. Remarkably, after longer reaction times, dichlorocarbene (**1a**) generated from chloroform, removed sulfur from both thiiranes *cis*-**3aa**, and *trans*-**3aa** yielding bis-ethylene derivative **4aa** (Scheme 1) [24].

**Scheme 2:**
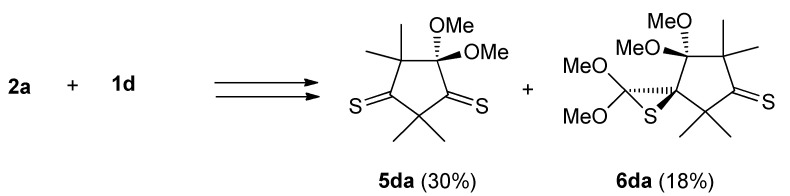
Products obtained in reactions of 2,2,4,4-tetramethylcyclobutane-1,3-dithione (**2a**) with nucleophilic dimethoxycarbene (**1d**).

On the other hand, the same thioketone **2a** reacted with nucleophilic dimethoxycarbene (**1d**) yielding ring-expanded cyclopentanedithione **5da** and thiirane **6da**, derived from the latter compound, were isolated as major products (Scheme 2) [25].

Whereas theoretical studies on mechanisms of [2 + 1] cycloadditions of carbenes with ethylenic compounds are widely discussed in numerous publications [1,2,3,26,27,28], there is a lack of similar works focused on reactions with thiocarbonyl compounds. Appearance of 1,3-dipoles such as azomethine ylides, carbonyl ylides, or thiocarbonyl ylides as reactive intermediates in reactions of carbenes/carbenoiods with imines [29,30], carbonyl [31], and thiocarbonyl compounds [32,33], respectively, has been discussed in numerous publications but the details of postulated mechanisms have never been elucidated using theoretical methods. Due to our continuous interest in the chemistry of thiocarbonyl compounds on one side and in nucleophilic as well as electrophilic carbenes on the other one, we decided to study reactions of some, synthetically significant thioketones with both types of carbenes in detail, and the DFT method was selected as an appropriate tool to solve this problem.

The goal of the present computational study was elucidation of the nature of reactive intermediates, which appear in the course of reactions of non-enolizable, sterically crowded thioketones **2a–c**, derived from 2,2,4,4-tetramethylcyclobutanone, as well as 2,2,5,5-tetramethylcyclopentanethione (**2d**) and disclosure of differences between reactions pathways observed in its reactions with differently substituted, electrophilic and nucleophilic carbenes (**1a–b** versus **1c–d**).

## 2. Results and Discussion

The presented study is divided into three parts: (i) firstly, the analysis of the electronic properties of addends and their intermolecular interactions according to Conceptual Density Functional Theory (CDFT) reactivity indices is carried out; (ii) in the next paragraph, a full mechanistic consideration of the reaction of dichlorocarbene (**1a**) with 2,2,4,4-tetramethylcyclobutane-1,3-dithione (**2a**) is explored, characterized, and discussed; (iii) finally, selected aspects of reactions of carbenes **1a** and **1c**–**d** with thiones **2a**–**b** are studied.

### 2.1. Nature of Intermolecular Interactions in the Light of a the Analysis of the CDFT Reactivity Indices of Reagents

#### 2.1.1. Global Reactivity

The CDFT is considered a powerful tool for understanding the reactivity of substrates involved in a polar cycloaddition reaction. The CDFT indices were calculated at the B3LYP/6-31G(d) computational level in the gas phase, because it was used to define the universal electrophilicity and nucleophilicity scales [34,35]. The global reactivity indices, namely, electronic chemical potential μ, chemical hardness η, global electrophilicity ω, and global nucleophilicity N, for the reagents involved in reactions are given in Table 1.

Firstly, we analyzed interaction between addents reagarding the reaction of **2a** with **1a**. The electronic chemical potential [35,36] µ of **2a**, −4.24 eV, is significantly higher than this of **1a**, −5.45 eV (Table 1). It means that, in the course of reaction **1a** + **2a,** the flux of the electron density will take place from thione **2a** to carbene **1a**. A similar situation is observed for reactions between thioketones **2b**–**d** and carbene **1a** (Table 1). Moreover, the electronic chemical potential µ of dibromocarbene (**1b**), −5.33 eV, is significantly lower than these of **2a**–**d** (Table 1). It means that, in the course of reaction of **1b** with **2a**–**d** the flux of the electron density will be directed also from thioketones **2a**–**d** to carbene **1b**. On the other hand, electronic chemical potential µ of difluorocarbene (**1c**) and dimethoxycarbene (**1d**), −3.00 and −2.90 eV, respectively, is significantly higher than in analyzed thiones **2a**–**d** (Table 1). It means that, for the reaction **1c** + **2a**–**d**, and also **1d** + **2a**–**d**, the flux of electron density will be directed from carbenes **1c** and **1d** to thioketones **2a**–**d**.

The calculated electrophilicity [35] ω index of thioketones **2a**–**d** are equal to 2.58, 2.35, 1.87, and 1.84 eV, respectively. On the other hand, the nucleophilicity [37] indexes N for these compounds are equal to 3.14, 3.09, 3.35, and 3.37 eV, respectively (Table 1). These values allow to classify thioketones **2a**–**d** as strong nucleophiles and also strong electrophiles within the corresponding electrophilicity and nucleophilicity scales [34,35,36,37]. Consequently, thioketones **2a**–**d** will behave as typical ambiphilic species [37]. Nevertheless, dithioketone **2a** and monothioketone **2b** are characterized by stronger electrophilic properties in comparison with thioketones **2c** and **2d**.

The electrophilicity ω indexes of dichlorocarbene (**1a**) and dibromocarbene (**1b**) were calculated to 3.91 and 4.11 eV, respectively. It means that both carbenes **1a** and **1b** can be classified as superelectrophilic species. On the other hand, the nucleophilicity N indexes of **1a** and **1b** were calculated to 1.76 and 2.06 eV, respectively (Table 1). Therefore, they can be classified as a marginal nucleophiles.

In turn, the electrophilicity ω indexes of difluorocarbene (**1c**) and dimethoxycarbene (**1d**) were calculated to 0.81 and 0.75 eV, respectively, and these values are drastically lower in comparrison to electrophilicity ω index of **1a** and **1b**. On the other hand, the nucleophilicity N indexes of **1c** and **1d** were calculated to 2.94 and 3.42 eV, respectively, and these values are relatively higher when compared with the nucleophilicity N index of **1a** and **1b** (Table 1). In consequence, carbenes **1c** and **1d** can be classified as marginal electrophiles but strong nucleophile at the same time.

In summary, for reactions between thioketones **2a**–**d** with dichlorocarbene (**1a**) and dibromocarbene (**1b**), all thioketones **2a**–**d** will act as nucleophilic species (electron donors), whereas carbenes **1a** and **1b** will behave as electrophiles (electron acceptors). In contrast, for reactions involving thioketones **2a**–**d** and difluorocarbene (**1c**) or dimethoxycarbene (**1d**), all **2a**–**d** will play the role of electrophiles (electron acceptors), while carbenes **1c** and **1d** will participate as nucleophilic reagents (electron donors).

#### 2.1.2. Local Reactivity

**Figure 2:**
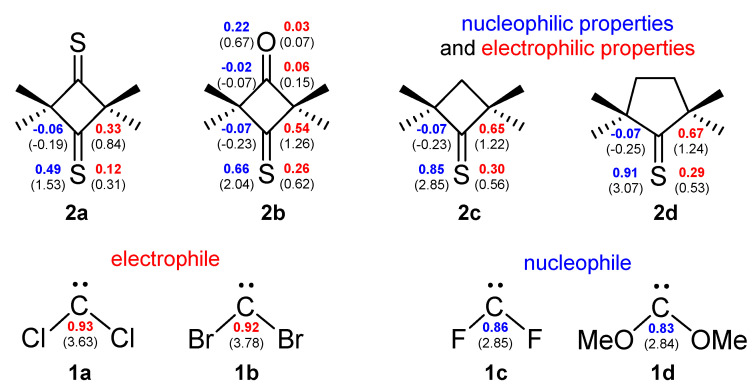
The local electronic properties of carbenes **1a**−**d** and thioketones **2a**−**d**. The nucleophilic P_k_^−^ given in blue and the electrophilic P_k_^+^ given in red; the indexes of local nucleophilicity N_k_ and local electrophilicity ω_k_ given in brackets.

The regioselectivity of polar processes of non-symmetric reagents can be specified through interaction between the most electrophilic center of the electrophile and the most nucleophilic ones of the nucleophile. In this context, the electrophilic P_k_^+^ and nucleophilic P_k_^−^ Parr functions were shown to be the most accurate and insightful tools for the study of local reactivity [38]. In order to characterize the most nucleophilic and the most electrophilic center of the species involved in the analyzed reactions, the electrophilic P_k_^+^ and nucleophilic P_k_^−^ Parr functions of carbenes **1a**–**d** and thioketones **2a**–**d** were studied (Figure 2).

Analysis of the nucleophilic P_k_^−^ Parr functions of thioketones **2a** and **2c**–**d** indicates that the sulfur atom of >C=S fragment constitutes the most nucleophilic center of these species presenting the maximum values at P_k_^−^ = 0.49 (**2a**), 0.85 (**2c**), and 0.91 (**2d**) eV, respectively (Figure 2). On the other hand, analysis of the electrophilic P_k_^+^ Parr functions of thiones **2a** and **2c**–**d** indicates that the carbon atom of >C=S fragment is the most electrophilic center of these species presenting the maximum values at P_k_^+^ = 0.33 (**2a**), 0.65 (**2c**) and 0.67 (**2d**) eV, respectively (Figure 2). Moreover, the presence of two thiocarbonyl groups in **2a** results in a significant reduction of values for both local nucleophilicity and local electrophilicity. On the other hand, 3-thioxocyclobutanone **2b** possesses two significant reactive centers, namely >C=S and >C=O groups. Therefore, the nucleophilic P_k_^−^ Parr functions of thioketone **2b** indicates that the sulfur atom constitutes the most nucleophilic center, presenting the maximum value at P_k_^−^ = 0.66 eV. In turn, the electrophilic P_k_^+^ Parr functions of **2b** indicates that the carbon atom of >C=S fragment is the most electrophilic center, presenting the maximum value at P_k_^−^ = 0.54 eV (Figure 2). Notably, in comparison to **2c** and **2d**, the presence of >C=S and >C=O moieties in the structure of **2b** does not result in significant changes in values of local nucleophilicity and local electrophilicity, as well. It is worth mentioning that all of analyzed thioketones **2a**–**d** display a similar reactivity.

According to CDFT theory [34,39], the described interactions show that the reactions of thioketones **2a**–**d** with dichlorocarbene (**1a**) and dibromocarbene (**1b**) in the initial phase will be realized through the interaction of sulfur atom of **2a–d** with the two-valent carbon atom of carbenes **1a**–**b**. In turn, reactions of **2a**–**d** with difluorocarbene (**1c**) and dimethoxycarbene (**1d**) are initiated by the interaction of carbon atom of the C=S group with reactive C-center of respective carbene.

### 2.2. A Full Mechanistic Considerations Reaction of Dichlorocarbene (***1a***) with 2,2,4,4-Tetramethylcyclobutane-1,3-dithione (***2a***)

**Scheme 3:**
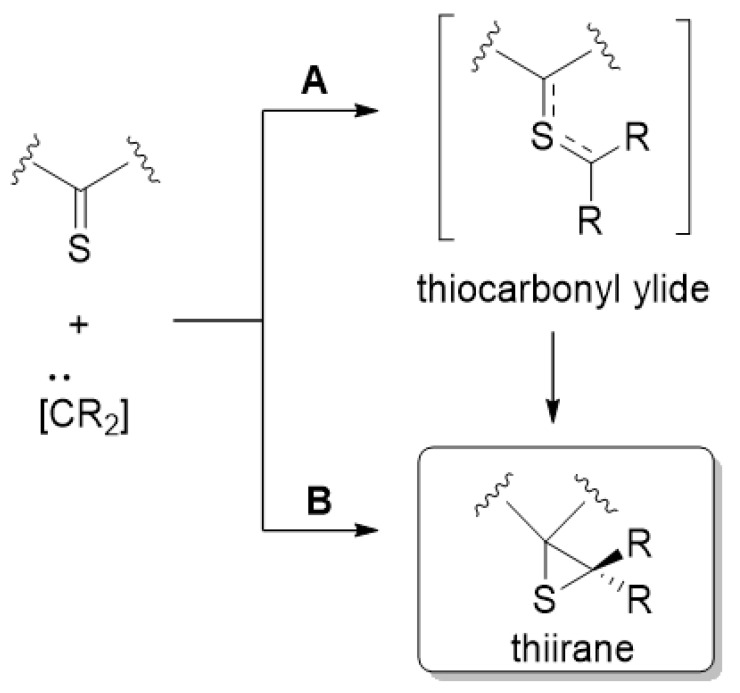
Plausible pathways for the formation of the thiirane ring in reactions of thioketones with carbene species.

For reactions of thioketone and with carbene species, two competitive mechanisms can be considered (Scheme 3). In the first case (route **A**), the formation of the thiirane ring occurs via stepwise mechanism, with the intervention of thiocarbonyl ylide as a reactive intermediate [40]. Alternatively (route **B**), the thiirane ring can be formed according to the one-step [2 + 1] cycloaddition scheme [26,41].

**Figure 3:**
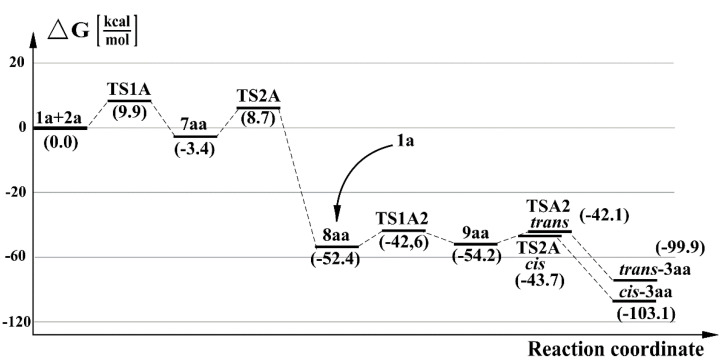
Gibbs free energy profile for the reaction of dichlorocarbene (**1a**) with 2,2,4,4-tetra-methylcyclobutane-1,3-dithione (**2a**) in the light of wb97xd/6-311g(d,p)(PCM) computational study.

The computational wb97xd/6-311g(d,p)(PCM) study was performed based on the model reaction of dichlorocarbene (**1a**) with 2,2,4,4-tetramethylcyclobutane-1,3-dithione (**2a**). Obtained results demonstrated that this transformation, carried out in the chloroform solution, occurs via stepwise mechanism [42], with the participation of two transition states and a single intermediate (Figure 3).

In the first step, the reaction occurs via **TS1A** transition state, and its formation is accompanied by an increase of the Gibbs free energy of the reaction system of ca. 10kcal/mol. Within this TS, a single new 𝕾 bond is formed between C(1) and S(2) reaction sites. At the same time, the C(1)-C(3) distance remains beyond of the range that is typical for C-C bonds in the transition state [43]. Subsequently, the IRC analysis connects the energetic maximum of TS, with the valleys of individual reagents **1a** + **2a** and **7aa** intermediate. So, the localized TS should be connected with the **A**-type reaction mechanism (Scheme 3). The further movement from the TS area along the reaction coordinate leads to the minimum connected with the existence of thiocarbonyl ylide **7aa**. Evidently, the reaction is determined by an attack of carbene species onto the sulfur atom of the >C=S moiety, which correlates excellently with the nature of local interactions analyzed within the previous paragraph.

From an energetic point of view, addition of **1a** to **2a** is favorable from thermodynamic point of view, because the Gibbs free energy of thiocarbonyl ylide **7aa** is substantially lower than individual addents **1a** and **2a**. Within the molecule of transient thiocarbonyl ylide, the central >C=S=C< fragment displays the planar structure, and it is located coplanar to four-membered cyclobutane ring. The transformation of **7aa** into **8aa** is realized via single TS (**TS2A**) and this process is accompanied by the overcoming of the activation barrier calculated to ca. 12 kcal/mol. Within **TS2A**, the interatomic distance C(1)-C(3) is shortened when compared with analogous distance in transient ylide **7aa**. Subsequently, the dihedral angle between the >C=S=C< fragment and the cyclobutane ring is increased to 48°. The IRC calculations connect this TS with valleys of **8aa** and **9aa**. The **8aa**→ **9aa** is fully irreversible, because the Gibbs free energy of the transformation is equal to −49kcal/mol. Within the **9aa** structure, the three-membered ring is fully formed, and it is perpendicularly located to the plane of cyclobutane ring.

**Figure 4:**
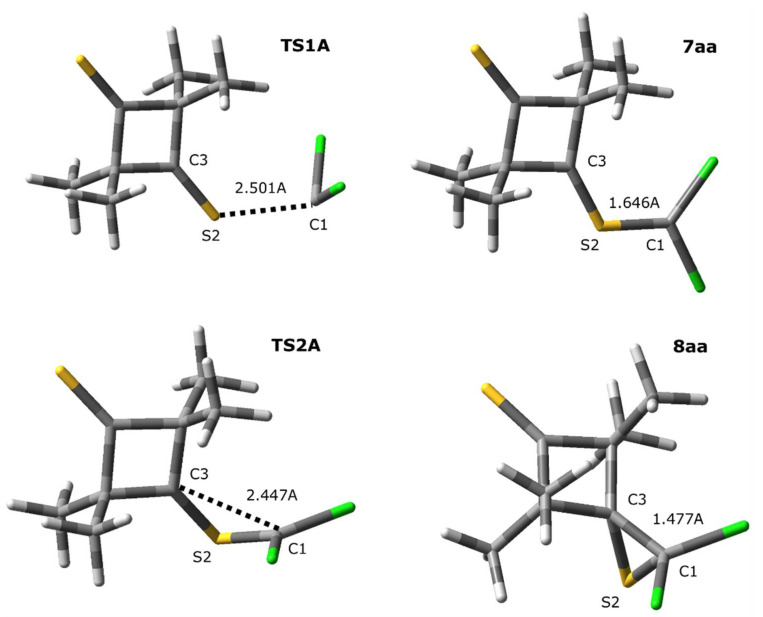
Views of key structures for **1a** + **2a** → **8aa** transformation, in the reaction starting with carbene **1a** and thione **2a** in the light of wb97xd/6-311g(d,p)(PCM) computational study.

**Figure 5:**
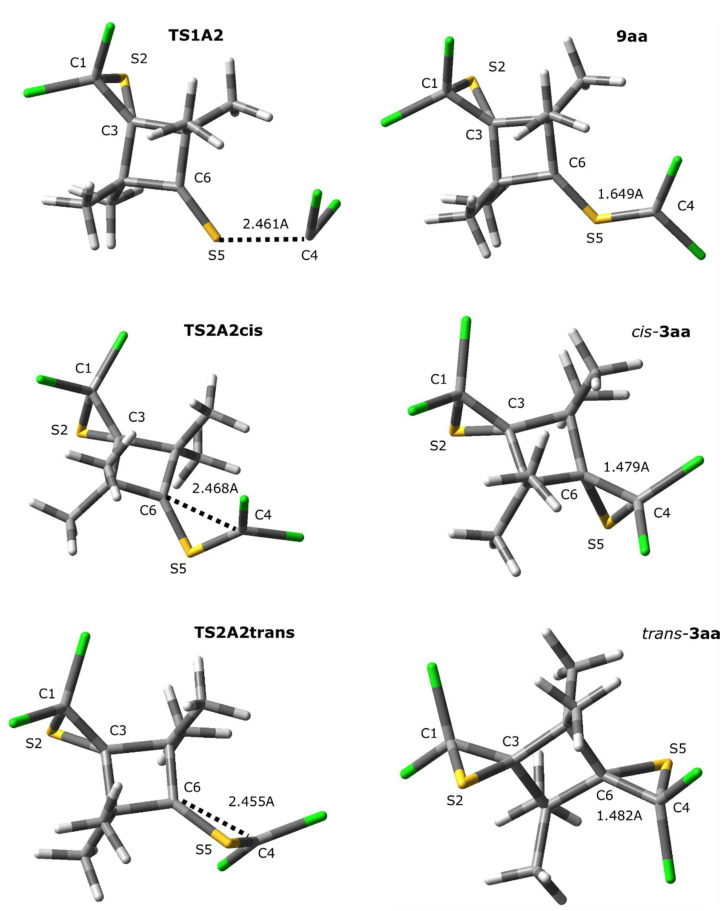
Views of key structures for **8aa**→*cis*-**3aa**/*trans*-**3aa** transformation in the reaction starting with carbene **1a** and thioketone **2a** in the light of wb97xd/6-311g(d,p)(PCM) computational study.

The existence of the second >C=S bond in the starting molecule determines two-fold addition of carbene, which finally leads to the formation of a mixture of stereoisomeric bis-adducts, i.e., *cis*-**3aa** and *trans*-**3aa**. The computational study demonstrated that both transformations: **8aa + 1a** → *cis*-**3aa** and **8aa + 1a** → *trans*-**3aa** are realized via analogous mechanism as in the case of already discussed reaction **1a + 2a** → **8aa** (Figure 4 and Figure 5).

**Scheme 4:**
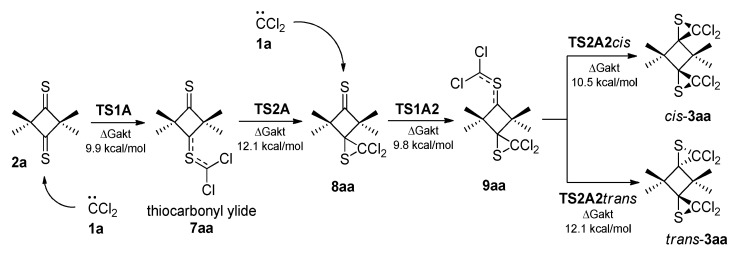
Mechanistic scheme for two-fold addition of **2a** to **1a** in the light of wb97xd/6-311g(d,p)(PCM) computational study.

At this point, it should be underlined that the more favored kinetical channels of the cyclisation of **9aa** proceed via **TS2A2** *cis* transition state and lead to formation of bis-*spiro*-adducts with *cis* configuration of heterocyclic rings in the target molecule. General, mechanistic scheme of addition of dichlorocarbene (**1a**) with 2,2,4,4-tetramethyl-cyclobutane-1,3-dithione (**2a**) is presented in Scheme 4.

### 2.3. Further Aspects of Reactions between Carbenes ***1a***–***d*** and Thioketones ***2a***–***d***

**Figure 6:**
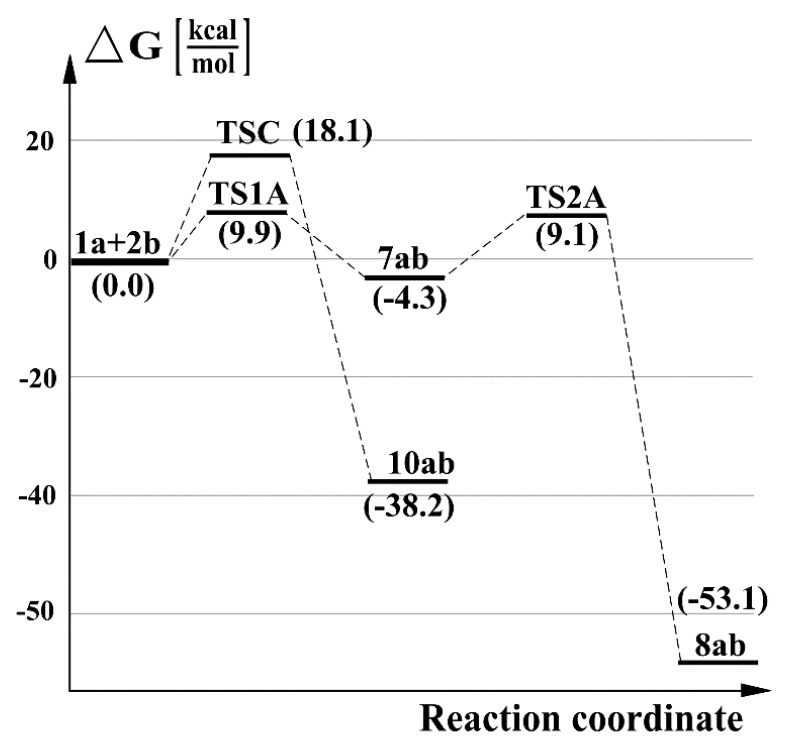
Gibbs free energy profile for reaction of dichlorocarbene (**1a**) with 3-thioxo-2,2,4,4-tetra-methylcyclobutanone (**2b**) with in the light of wb97xd/6-311g(d,p)(PCM) computational study.

According to a similar scheme proceed analogous reactions involving other thioketone **2** (Tables S1–S5 in Supplementary Materials). It is worth underscoring that in the case of monothioketone **2b**, reaction theoretically can proceed upon involvement of >C=S, and/or >C=O bond. The analysis of the local reactivity suggests substantially higher reactivity of the first unit. Indeed, full exploration of reaction channels with the localization, optimization, and verification of all critical structures confirmed this hypothesis. In particular, the wb97xd/6-311g(d,p)(PCM) calculations show that the reaction on the >C=S bond requires the Gibbs free energy of the activation step of ca. 10kcal/mol, whereas the competitive process involving >C=O bond—more than 18kcal/mol (Figure 6).

**Scheme 5:**
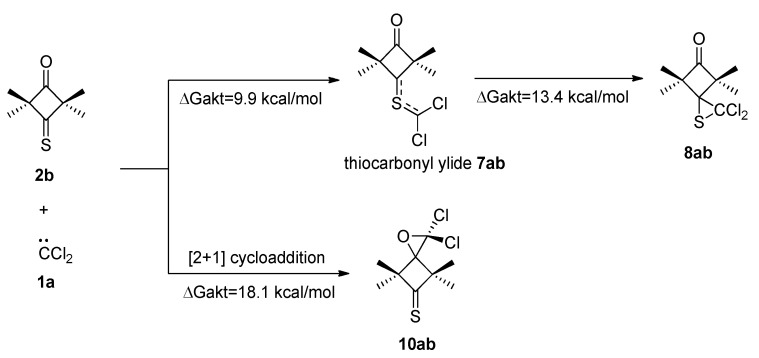
Mechanistic scheme for **1a** + **2b** reaction in the light of wb97xd/6-311g(d,p)(PCM) computational study.

**Figure 7:**
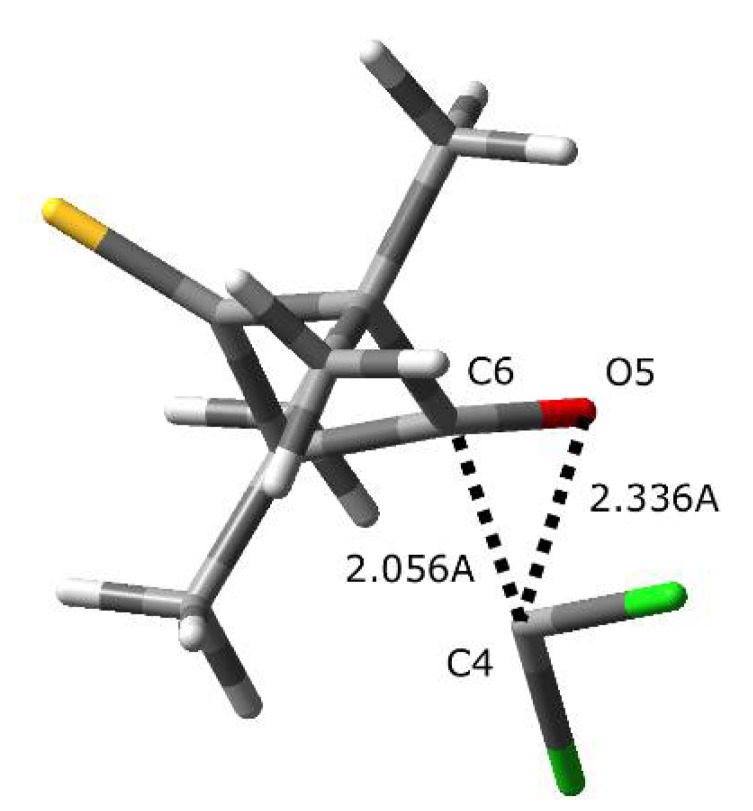
Views of the TS structure for **1a + 2b**→**10ab** transformation in the reaction starting with carbene **1a** and thioketone **2b** in the light of wb97xd/6-311g(d,p)(PCM) computational study.

Consequently, from a kinetic point of view, within the analyzed reaction system, the addition of **1a** to the >C=O moiety, leading to an intermediate carbonyl ylide, should be considered as a forbidden process. From a mechanistic point of view, thus, the reaction leading to 2,2-dichlorooxirane derivative **10ab**, proceeds via a completely different scheme as in the case of thiirane formation. In particular, the addition occurs via single transition state. Within this TS, two new 𝕾 bonds are formed simultaneously and formally, this process can be considered as the [2 + 1] cycloaddition reaction (Figure 7). Generally, the mechanistic scheme of addition of dichlorocarbene (**1a**) to 3-thioxo-2,2,4,4-tetramethylcyclobutanone (**2b**) is presented in Scheme 5.

**Scheme 6:**
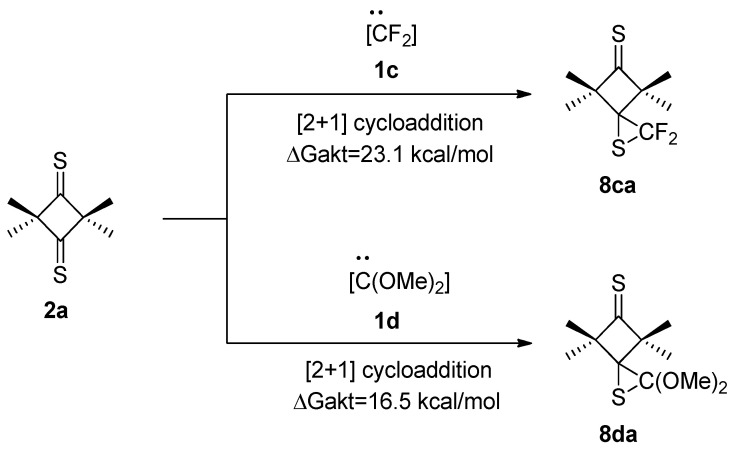
Mechanistic scheme for **2a** + **1c/1d** reactions in the light of wb97xd/6-311g(d,p)(PCM) computational study.

Finally, additions of nucleophilic carbenes **1c** and **1d** to thioketone **2a** were also analyzed (Scheme 6).

In contrast to transformation involving carbene **1a**, this reaction proceeds via single transition state (**TSB**). This process is accompanied by the overcome of the activation barrier of 23.1 kcal/mol and 16.5 kcal/mol, respectively, for reactions involving less nucleophilic difluorocarbene (**1c**), and more nucleophilic dimethoxycarbene (**1d**). The kinetic aspects of considered additions correlate perfectly with the nature of global interaction between addents. Within the discussed TS, two new 𝕾 bonda are formed; firstly, between C(1) and S(2) and secondly, between C(1) and C(3) atoms. Thus, formally, this process can be interpreted as the [2 + 1] cycloaddition reaction. The IRC analysis confirms that the localized TS can directly connect with respective valleys of reagents and heterocyclic adducts.

Earlier, experimental studies demonstrated that thiirane **8cd** is efficiently formed from **1c** and **2d** (generated from Seyerth’s regaent) [23]. However, in the case of **2a** and **1d**, expected thiirane **8da** was a minor product, only, and nucleophilic **1d** preferentially attacked congested cyclobutane ring yielding five-membered, ring-expanded products [25]. Nevertheless, reaction of in situ generated **1d** with non-congested adamantenthione yielded corresponding 2,2-dimethoxythiirane as final product, isolated in high yield (83%) [44].

## 3. Computational Details

All quantumchemical calculations were performed using ‘Prometheus’ cluster (CYFRONET regional computational center).

The global reactivity indices of the reactants, namely electronic potential μ, chemical hardness η, global electrophilicity ω, and global nucleophilicity N, were estimated in pursuance of the equations defined on the basis of conceptual density functional theory (CDFT) according to the equations recommended by Parr [35] and Domingo [34,45]. In the calculation are used the correlation-exchange functional B3LYP together with the basis 6-31G(d) level set in the gas phase. In this section, the B3LYP/6-31g(d) approach was applied because the electrophilicity and nucleophilicity scales that allow classification of molecules based on their electrophilic and nucleophilic character were established at that level [34,39,45,46].

The electronic chemical potentials (μ) and chemical hardness (η) were evaluated in terms of one-electron energies of FMO (E_HOMO_ and E_LUMO_) using the following equations [35,46]:

$$\mu \approx \frac{E_{\mathrm{HOMO}}+{\;E}_{\mathrm{LUMO}}}{2}$$

$$\eta \approx E_{\mathrm{HOMO}}-E_{\mathrm{LUMO}}$$

where E_HOMO_ and E_LUMO_ may be approached in terms of the one-electron energies of the frontier MOs respectively HOMO and LUMO.

The values of chemical potentials (μ) and chemical hardness (η) were then used to calculate a global electrophilicity (ω) according to the formula [34,35,47]:

$$\omega =\frac{\mu^{2}}{\eta }$$

In turn, the global nucleophilicity (N) can be expressed as [45]:

$$N=E_{\mathrm{HOMO}}-E_{\mathrm{HOMO}\;\left(\mathrm{TCE}\right)}$$

where E_HOMO (TCE)_ is the HOMO energy for tetracyanoethylene (TCE); is the reference, because it presents the lowest HOMO (E_HOMO (TCE)_ = –9.368 eV).

The local electrophilicity (ω_k_) and the local nucleophilicity (N_k_) concentrated on atom k was calculated based on global properties and the Parr function (P_k_^+^ or P_k_^−^), according to the formulas [38]:

$$\omega_{k}=P_{k}^{+}\cdot \omega$$

$$N_{k}=P_{k}^{-}\cdot N$$

For the simulation of the reactions paths, the wb97xd functional included in the GAUSSIAN 09 package [48] and the 6-311g(d,p) basis set [49,50] including both diffuse and polarization functions for all relevant atoms was used. A similar theory level has been commonly used for the mechanistic research aspects of cycloaddition reactions [28,39,51,52,53,54,55]. All localised stationary points have been characterized using vibrational analysis. It was found that starting molecules as well as products had positive Hessian matrices. On the other hand, all transition states (**TS**) showed only one negative eigenvalue in their Hessian matrices. For all optimized transition states, intrinsic reaction coordinate (IRC) calculations have been performed. The solvent effect has been included using PCM algorithm [56].

## 4. Conclusions

The presented computational study demonstrated that additions of dihalocarbenes to non-enolisable, cycloaliphatic thioketones proceed differently and depend on the type of carbene species involved in the reaction. Whereas electrophilic dichlorocarbene (**1a**) and dibromocarbene (**1b**) attack the sulfur atom forming thiocarbonyl ylides as reactive intermediates, less electrophilic difluorocarbene (**1c**) tend to undergo one-step (but asynchronous) [2 + 1] cycloaddition leading to respective 2,2-difluorothiirane. In analogy to **1c**, reaction pathway, without formation of an intermediate thiocarbonyl ylide, is observed in the reaction of dimethoxycarbene (**1d**), which is considered as a typical representative of non-heterocyclic, nucleophilic carbenes.

In contrast to non-halogenated thiocarbonyl ylides postulated dichloro and dibromo functionalized intermediates do not undergo secondary processes such as [3 + 2] cycloaddition to >C=S bond leading to 1,3-dithiolanes [57] or [3 + 3] ‘head to head‘ dimerization yielding 1,4-dithianes [58].

Obtained results allow us to better understand mechanisms of reactions of carbenes with thiocarbonyl compounds leading to thiiranes or products of their secondary conversions [40]. In addition, they nicely complement experimental observations on appearance of reactive thiocarbonyl *S*-methanides (thiocarbonyl ylides) in reactions of non-enolizable thioketones with electrophilic methylene [:CH_2_] carried out in low-temperature Ar-matrices [59].

## Figures and Tables

**Table 1:** Global electronic properties (electronic chemical potential μ, chemical hardness η, global electrophilicity ω, and nucleophilicity N; all in eV) for the studied reagents.

	μ	η	ω	N
**1a**	−5.45	3.81	3.91	1.76
**1b**	−5.33	3.46	4.11	2.06
**1c**	−3.00	5.60	0.81	3.32
**1d**	−2.90	5.59	0.75	3.42
**2a**	−4.24	3.49	2.58	3.14
**2b**	−4.17	3.71	2.35	3.09
**2c**	−3.82	3.90	1.87	3.35
**2d**	−3.80	3.91	1.84	3.37

## Data Availability

The data presented in this study are available on request from the corresponding author.

## Supplementary Materials

The following are available online. Acronyms and abbreviations, computational details, Tables S1–S26.

## Sample Availability

Samples of the compounds are not available from the authors.
