# Supplementary material for: A DFT Study on the Molecular Mechanism of Additions of Electrophilic and Nucleophilic Carbenes to Non-Enolizable Cycloaliphatic Thioketones
Source: Molecules. 2021 Sep 13;26(18):5562. doi: 10.3390/molecules26185562 (PMC8466156; doi:10.3390/molecules26185562)
Supplement: Supplementary file 1 [file molecules-26-05562-s001.zip › molecules-1365986-supplementary.pdf]

molecules

MDPI

SUPPLEMENTARY MATERIALS

A DFT study on the molecular mechanism of additions of electrophilic and nucleophilic carbenes to non-enolizable cycloaliphatic thioketones

Grzegorz Mlostoń [1,*], Karolina Kula [2], Radomir Jasiński [2,*]

[1] Department of Organic and Applied Chemistry
University of Łódź, Tamka 12, PL-91-403 Łódź, Poland
grzegorz.mloston@chemia.uni.lodz.pl

[2] Institute of Organic Chemistry & Technology
Cracow University of Technology
Warszawska 24, PL-31-155 Krakow, Poland
kkula@chemia.pk.edu.pl (K.K.)
radomir@chemia.pk.edu.pl (R.J.)

INDEX

3 **Table S1 –** Eyring parameters of reactions carbene **1a** and thione **2a** (wb97xd/ 6-311g(d,p)(PCM), chloroform).

3 **Table S2 –** Eyring parameters of reactions thione **1a** and thione **2b** (wb97xd/ 6-311g(d,p)(PCM), chloroform).

3 **Table S3 –** Eyring parameters of reactions carbene **1a** and thione **2c** (wb97xd/ 6-311g(d,p)(PCM), chloroform).

4 **Table S4 –** Eyring parameters of reactions carbene **1a** and thione **2d** (wb97xd/ 6-311g(d,p)(PCM), chloroform).

4 **Table S5 –** Eyring parameters of reactions thione **2a** and carbenes **1b-d** (wb97xd/6-311g(d,p)(PCM), chloroform).

4 **Table S6 –** Selected parameters of key structures for carbenes **1a-d** (wb97xd/ 6-311g(d,p)(PCM), chloroform).

5 **Table S7** and **S8 –** Selected parameters of key structures for reaction between carbene **1a** and thione **2a** (wb97xd/6-311g(d,p)(PCM), chloroform).

5 **Table S9** and **S10 –** Selected parameters of key structures of reaction between carbene **2a** and thione **1b** (wb97xd/6-311g(d,p)(PCM), chloroform).

6 **Table S11 –** Selected parameters of key structures for reaction between carbene **2a** and thiones **1c-d** (wb97xd/6-311g(d,p)(PCM), chloroform).

6 **Table S12 –** Selected parameters of key structures of reaction between carbenes **1b-d** and thione **2a** (wb97xd/6-311g(d,p)(PCM), chloroform).

Table S1. Kinetic and thermodynamic parameters of reaction between carbene **1a** and thione **2a** in chloroform solution, according to wb97xd/6-311g(d,p)(PCM) calculations (ΔH and ΔG values are giving in kcal/mol^{-1}; ΔS values are giving in cal/mol^{-1} K^{-1}).

Transition	ΔH	ΔG	ΔS
**1a + 2a → TS1A**	-2.0	9.9	-39.8
**1a + 2a → 7aa**	-15.9	-3.4	-41.9
**7aa → TS2A**	10.3	12.1	-6.0
**7aa → 8aa**	-51.6	-49.0	-8.7
**8aa + 1a → TS1A2**	-2.1	9.8	-39.7
**8aa + 1a → 9aa**	-14.3	-1.8	-42.0
**9aa → TS2A2*cis***	8.9	10.5	-5.2
**9aa → *cis*-3aa**	-51.0	-48.9	-7.3
**9aa → TS2A2*trans***	10.5	12.1	-5.4
**9aa → *trans*-3aa**	-48.7	-45.7	-9.8

Table S2. Kinetic and thermodynamic parameters of reaction between carbene **1a** and thione **2b** in chloroform solution, according to wb97xd/6-311g(d,p) (PCM) calculations (ΔH and ΔG values are giving in kcal/mol^{-1}; ΔS values are giving in cal/mol^{-1} K^{-1}).

Transition	ΔH	ΔG	ΔS
**1a + 2b → TS1A**	-2.2	9.9	-40.5
**1a + 2b → 7ab**	-16.8	-4.3	-41.7
**7ab → TS2A**	11.2	13.4	-7.3

**7ab → 8ab**	-51.2	-48.8	-8.1
**1a + 2b → TSC**	5.1	18.1	-43.5
**1a + 2b → 10ab**	-52.7	-38.2	-48.8

Table S3. Kinetic and thermodynamic parameters of reaction between carbene **1a** and thione **2c** in chloroform solution, according to wb97xd/6-311g(d,p)(PCM) calculations (ΔH and ΔG values are giving in kcal/mol^{-1}; ΔS values are giving in cal/mol^{-1} K^{-1}).

Transition	ΔH	ΔG	ΔS
**1a + 2c → TS1A**	-3.1	8.7	-39.4
**1a + 2c → 7ac**	-15.1	-3.4	-39.1
**7ac → TS2A**	9.1	11.8	-9.1
**7ac → 8ac**	-51.4	-47.7	-12.4

Table S4. Kinetic and thermodynamic parameters of reaction between carbene **1a** and thione **2d** in chloroform solution, according to wb97xd/6-311g(d,p) (PCM) calculations (ΔH and ΔG values are giving in kcal/mol^{-1}; ΔS values are giving in cal/mol^{-1} K^{-1}).

Transition	ΔH	ΔG	ΔS
**1a + 2d → TS1A**	-3.2	8.8	-40.4
**1a + 2d → 7ad**	-11.7	0.1	-39.6
**7ad → TS2A**	6.6	9.1	-8.4
**7ad → 8ad**	-47.3	-43.8	-11.6

Table S5. Kinetic and thermodynamic parameters of reaction between thione **2a** and carbenes **1b-d** in chloroform solution, according to wb97xd/6-311g(d,p) (PCM) calculations (ΔH and ΔG values are giving in kcal/mol^{-1}; ΔS values are giving in cal/mol^{-1} K^{-1}).

Transition	ΔH	ΔG	ΔS
**1b + 2a → TS1A**	-2.3	8.9	-37.4
**1b + 2a → 7ba**	-18.6	-6.7	-39.8
**7ba → TS2A**	10.5	12.6	-7.0
**7ba → 8ba**	-48.7	-45.7	-10.2
**1c + 2a → TSB**	10.2	23.1	-43.2
**1c + 2a → 8ca**	-53.6	-38.9	-49.5
**1d + 2a → TSB**	3.0	16.5	-45.2
**1d + 2a → 8da**	-53.8	-39.4	-48.6

Table S6. Selected parameters of carbenes **1a-d** (wb97xd/6-311g(d,p)(PCM), chloroform).

Structure	Fragment	Interatomic distances [Å]
**1a**	C—Cl	1.725
**1b**	C—Br	1.883
**1c**	C—F	1.299
**1d**	C—OMe	1.317

Table S7. Selected parameters of key structures of the reaction between carbene **1a** and thione **2a** to create **8aa** (wb97xd/6-311g(d,p)(PCM), chloroform).

Structure	Interatomic distances [Å]			GEDT
	C1-S2	S2-C3	C1-C3	[e]
**2a**		1.611		
**TS1A**	2.501	1.615	3.638	0.287
**7aa**	1.646	1.623	2.808	
**TS2A**	1.685	1.679	2.447	0.000
**8aa**	1.785	1.844	1.478	

Table S8. Selected parameters of key structures of the reaction between thione **8aa** and carbene **1a** to create *cis*-**3aa** and *trans*-**3aa** (wb97xd/6-311g(d,p)(PCM), chloroform).

Structure	Interatomic distances [Å]			GEDT
	C4-S5	S5-C6	C4-C6	[e]
**8aa**		1.612		
**TS1A2**	2.461	1.617	3.631	0.309
**9aa**	1.650	1.626	2.824	
**TS2A2***cis*	1.684	1.681	2.468	0.000
cis-**3aa**	1.783	1.849	1.479	
**TS2A2***trans*	1.685	1.679	2.455	0.000
trans-**3aa**	1.781	1.849	1.482	

Table S9. Selected parameters of key structures of the reaction between carbene **1a** and thione **2b** to create **7ab** (wb97xd/6-311g(d,p)(PCM), chloroform).

Structure	Interatomic distances [Å]			GEDT
	C1-S2	S2-C3	C1-C3	[e]
**2b**		1.612		
**TS1A**	2.545	1.615	3.678	0.265
**7ab**	1.647	1.622	2.805	
**TS2A**	1.685	1.679	2.443	0.000
**8ab**	1.786	1.846	1.476	

Table S10. Selected parameters of key structures of the reaction between thione **8ab** and carbene **1a** to create **10ab** (wb97xd/6-311g(d,p)(PCM), chloroform).

Structure	Interatomic distances [Å]			GEDT
	C4-O5	O5-C6	C4-C6	[e]
**8ab**		1.196		
**TSB**	2.336	1.231	2.056	0.024
**10ab**	1.365	1.447	1.454	

Table S11. Selected parameters of key structures of the reaction between carbene **1a** and thiones **2c-d** and (wb97xd/6-311g(d,p)(PCM), chloroform).

Thioketone	Structure	Interatomic distances [Å]			GEDT
		C1-S2	S2-C3	C1-C3	[e]
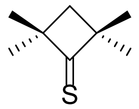 **2c**	**2c**		1.618		
	**TS1A**	2.706	1.619	3.853	0.191
	**7ac**	1.649	1.626	2.824	
	**TS2A**	1.682	1.690	2.460	0.000
	**8ac**	1.781	1.857	1.478	
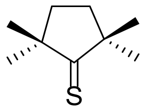 **2d**	**2d**		1.628		
	**TS1A**	2.599	1.633	3.881	0.230
	**7ad**	1.652	1.632	2.892	
	**TS2A**	1.678	1.703	2.457	0.000
	**8ad**	1.774	1.878	1.493	

Table S12. Selected parameters of key structures of the reaction between carbenes **1b-d** and thione **2a** and (wb97xd/6-311g(d,p)(PCM), chloroform).

Carbene	Structure	Interatomic distances [Å]			GEDT
		C1-S2	S2-C3	C1-C3	[e]
$\ddot{C}Br_2$ **1b**	**2a**		1.611		
	**TS1A**	2.645	1.613	3.786	0.187
	**7ba**	1.648	1.622	2.829	
	**TS2A**	1.688	1.679	2.446	0.000

	**8ba**	2.446	1.843	1.480	
$\ddot{C}F_2$ **1c**	**2a**		1.611		
	**TSB**	2.795	1.659	2.152	0.107
	**7ca**	1.783	1.861	1.460	
$\ddot{C}(OMe)_2$ **1d**	**2a**		1.611		
	**TSB**	3.011	1.649	2.404	0.188
	**8da**	1.867	1.842	1.469	

The global electron density transfer (GEDT) values were designated based on the formula:

GEDT = + ΣqA

where qA is the net Mulliken charge, and the sum is performed over all the atoms of carbenes **1a-d**.
